# Microbial Assessment and Antibiotic Susceptibility of Isolated Pathogens in Retail Chicken

**DOI:** 10.3390/foods14101738

**Published:** 2025-05-14

**Authors:** Eniola Betiku, Philip Glen Crandall, Tomi Obe

**Affiliations:** 1 Department of Poultry Science, University of Arkansas System Division of Agriculture, Fayetteville, AR 72701, USA; ebetiku@uark.edu; 2 Department of Food Science, Center for Food Safety, University of Arkansas System Division of Agriculture, Fayetteville, AR 72704, USA; crandal@uark.edu

**Keywords:** *Salmonella*, *Campylobacter*, retail chicken, food safety, antimicrobial resistance, conventional, CON, raised without antibiotics, RWA

## Abstract

Poultry is U.S. consumers’ protein of choice with an annual consumption of nearly 45 kg per person. This increasing demand has required poultry producers to minimize pathogen contamination to protect public health. This study assessed *Salmonella* and *Campylobacter* incidence and loads in retail chicken from conventional (CON) and raised without antibiotics (RWA) sources, while profiling antibiotic resistance of selected isolates. A total of 170 chicken samples from two brands (A and B), including whole carcass WOG (60), parts (80), and giblets (30) were evaluated. Both pathogens were examined by culture and BAX^®^ system methods and confirmed isolates were identified. Aerobic bacteria count (AC), Enterobacteriaceae (EB), and lactic acid bacteria (LAB) were also tested using Petrifilms™. Selected isolates of *Salmonella* (22) and *Campylobacter* (24) were tested for antibiotic susceptibility using the Sensititre™ system. The overall respective incidence of *Salmonella* and *Campylobacter* was 36% and 35% with no difference between CON (33% and 25%) and RWA (23% and 29%), but product types differed (*p* < 0.05). *Salmonella* incidence was not different between the brands, but *Campylobacter* differed. Giblets had a higher incidence of both pathogens at 80% and 70%, respectively. The most and least abundant *Salmonella* serotypes were Infantis (60%) and Ouakam (2%), while *Campylobacter jejuni* was the abundant species. All the indicators differed (*p* < 0.05) between CON and RWA. Many isolated pathogens possessed resistance to at least one antibiotic, *Salmonella* (91%) and *Campylobacter* (38%), with multidrug resistance in 45% of CON and 36% of RWA *Salmonella* isolates. The highest resistance was to tetracycline and nalidixic acid for both pathogens and the lowest was to antibiotics in the macrolides class. These results highlight the need for robust microbial control at all levels, as both production practices showed notable contamination and antibiotic resistance, emphasizing the need for continued surveillance at the retail level and encouraging consumers to properly cook poultry to 165 °F.

## 1. Introduction

The U.S. poultry industry plays a major role in global poultry production, being the top broiler producer in the world [1]. Chicken continues to be the most consumed source of animal protein in the United States [2,3], thereby leading to a high demand for poultry products, especially skinless breast meat and wings which has led to a steady increase in production. A major challenge to poultry producers is ensuring microbial safety, because chicken, as a natural product, is frequently contaminated with foodborne pathogens, particularly *Salmonella* and *Campylobacter*. These pathogens pose a significant public health risk as the leading contributors to foodborne illnesses in the U.S., with raw chicken linked to about 1 million cases of foodborne illness annually [4]

Being a zoonotic pathogen, *Salmonella* can be transmitted to humans through the consumption of contaminated food products like poultry, meat, and eggs causing symptoms such as diarrhea, abdominal pain, and fever with potential long-term health problems like irritable bowel syndrome [5,6]. Globally, *Salmonella* has been attributed to causing about 93.8 million cases of gastroenteritis and 155,000 deaths annually [7], and between 1998 and 2022, the U.S. experienced a multi-state outbreak of salmonellosis linked to a chicken product, resulting in 187 illnesses and 42 hospitalizations [8]. Another significant poultry-related pathogen is *Campylobacter,* which is responsible for over 1.5 million cases of foodborne illness acquired domestically in the U.S. each year [9]. Like *Salmonella*, *Campylobacter* causes serious gastrointestinal issues with symptoms including diarrhea, abdominal pain, vomiting, and fever. *Campylobacter* infections can also lead to long-term health problems, such as Guillain–Barre syndrome, a serious nervous system condition [10]. Contamination by these two pathogens can occur at any point along the poultry supply chain, from the farm level, continuing through processing and distribution, and extending to retail outlets, where each step presents a unique risk for bacterial growth and contamination. Therefore, understanding the factors that contribute to microbial contamination is essential to improve food safety throughout the supply chain.

During poultry production, contamination can occur at different stages. At pre-harvest, contamination can happen on the farm where these pathogens can be introduced through the feed, water, litter, or contact with infected animals [11]. It is essential to effectively control foodborne pathogens at this level to minimize contamination during processing [12]. At post-harvest, where birds are slaughtered, the carcasses pass through different processing steps before packaging. This processing stage is crucial for controlling microbial contamination because sometimes it is the final control point before retail distribution. *Salmonella* and *Campylobacter* can also spread through contaminated equipment, processing surfaces, and workers, resulting in cross-contamination [13]. Once poultry products are at the retail level, additional handling like packaging, storage, and display conditions can affect pathogen levels in the packaged product. Regardless of the production system, poultry products must be handled carefully to prevent contamination and microbial growth before they reach the consumer. Factors like temperature abuse, storage practices, and retail environmental conditions can contribute to the rapid increase in the levels of pathogens in poultry products causing an increased risk of foodborne illness [14]. Moreover, if raw chicken is not properly handled or cooked by consumers, it increases the chance of a foodborne outbreak [15]. According to the CDC, one in every 25 retail chicken packages at the grocery store is contaminated with *Salmonella* [4]. Between 2009 and 2018, approximately 12% of outbreak-related illnesses in the United States were linked to chicken products [16]. This highlights the ongoing public health concern surrounding bacterial contamination and limiting potential for growth in retail chicken, emphasizing the need for strict food safety practices.

Poultry production systems, both conventional and non-conventional, can contribute to the prevalence and antimicrobial resistance of bacterial foodborne pathogens. Conventional poultry production usually involves raising birds in enclosed, environmentally controlled houses [17]. This method ensures high production efficiency, alongside the use of antibiotics for disease prevention, as well as carefully monitored feeding regimes [17,18]. In contrast, non-conventional systems such as organic, pastured, free-range, no antibiotics ever (NAE), and no antibiotics important to human medicine (NAIHM) systems have emerged in response to consumer demand for rearing methods perceived as more natural, healthier, and safer from foodborne pathogens [19]. Labels like NAE ensure that no antibiotics are used throughout the bird’s lifecycle [20], while NAIHM standards restrict the use of antibiotics that are critical for human health during production [21]. To better understand the implications of these vastly different production systems and the outcomes of microbial contamination, studies have explored the incidence of *Salmonella* and *Campylobacter* in conventional and non-conventional retail chicken. Their findings reveal mixed results with some studies reporting no significant differences in pathogen prevalence, while others suggest that non-conventional systems exhibited higher rates of bacterial contamination [22,23,24,25,26,27]. However, most of these studies were conducted several years ago with only a few recent studies available. Considering the recent rise in food product recalls, foodborne outbreaks, and illnesses, especially from 2024 to early 2025, there is a need for updated studies assessing microbial contamination at the retail level accounting for both production systems. Similarly, research on antibiotic resistance patterns have produced varying results, with some studies indicating higher resistance levels in conventionally raised poultry, while others find comparable or even greater resistance in non-conventional poultry [22,23,24,27]. These differences could be influenced by individual management practices, therapeutic antibiotic usage, and environmental conditions. All these management practices can play a role in the prevalence of foodborne pathogens and the development of antibiotic resistance. Nonetheless, there is a need for continued surveillance to assess food safety risks due to the incidence of pathogenic and spoilage organisms at the retail level. Considering non-conventional systems and their outdoor exposure, we hypothesized that retail chicken from these sources could exhibit higher microbial contamination unlike conventional systems where chickens are indoors on a commercial scale. Hence, this study investigated the incidence and loads of *Salmonella* and *Campylobacter* in retail chicken products from both conventional (CON) and non-conventional rearing operations (raised without antibiotics, RWA); then, the isolated pathogens were profiled for antibiotic resistance to provide valuable insights that could enhance consumer food safety.

## 2. Materials and Methods

### 2.1. Experimental Design and Sample Collection

**Figure 1:**
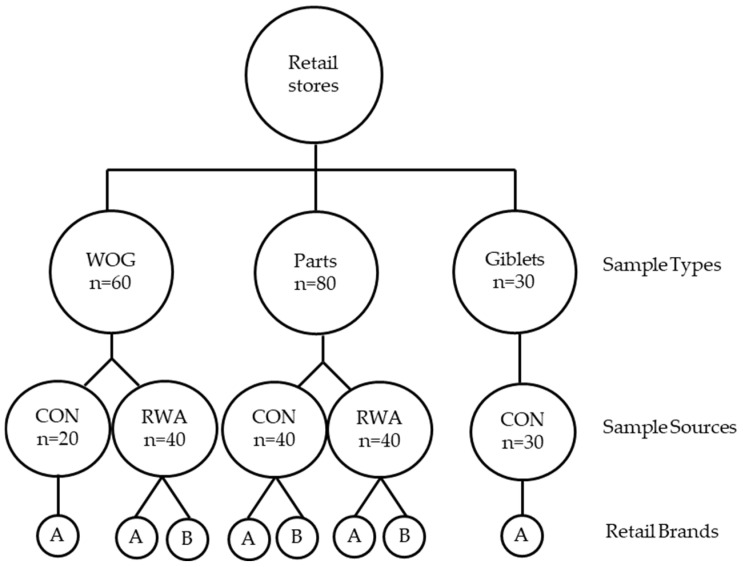
Schematic design of sample collection. Chicken sample types: whole carcass without giblets (WOG), chicken parts (drumstick and thigh), and giblets were collected at retail stores and categorized according to packaging information as conventional (CON) or raised without antibiotics (RWA) sources. The samples were collected from two retail brands (A and B). Twenty samples were collected from each brand based on availability for each sample type and source: WOG (60 samples: CON = 20 and RWA = 20 each brand), parts (80 samples: CON = 20 each and RWA = 20 each brand), and giblets (30 samples: CON only). Overall, 170 samples were collected.

Chicken products, consisting of whole carcass without giblets (WOG), chicken thigh and drumstick (parts), and giblets (liver, gizzard, and heart) from two production systems: CON and RWA, were obtained from various retail stores. The production systems were identified based on labelling claims. RWA products with USDA-verified labels such as Raised without antibiotics (RWA) and No Antibiotics Ever (NAE) were used. While the CON products used had no antibiotic-related claim. Before the experiment, a survey of different retail stores was conducted to determine the availability of different brands of chicken. Based on the survey and availability, for WOG, we identified only one brand (A) for CON and two brands (A and B) for RWA, whereas for parts, there were two brands each for CON and RWA. Giblets samples were available only from CON production system. A total of 170 chicken samples were collected from February to August 2024, with one of each chicken sample type collected on every sampling date. Ensuring that the “best by” dates were different for each sampling date helped to ensure that the samples were in fact from differing production lots. Our sample size, though small, aligns with other retail studies [24,25,26]. The sample distribution as shown in Figure 1 is as follows: (i) WOG (60 samples)-CON (A = 20) and RWA (brands A = 20 and B = 20), (ii) Parts (80 samples)-20 each for CON and RWA brands A and B, and (iii) giblets (30 CON samples). All samples were immediately transported to the laboratory for analysis while ensuring proper refrigeration conditions. All the media used in this study were purchased from VWR (VWR International Avantor^®^ Radnor, PA, USA) and Fisher Scientific (Hampton, NH, USA) unless otherwise specified.

### 2.2. Sample Processing

Isolation and detection of *Salmonella* and *Campylobacter* were conducted according to the USDA-FSIS MLG 4.15 and 41.08 methods, respectively [28]. In preparation for analysis, each retail chicken package was aseptically opened, and approximately 4 lbs. were placed in a large sterile rinse bag. Next, 400 mL of buffered peptone water (BPW) was added to each sample bag to make a rinsate. Each sample was then rinsed in and out vigorously for about one minute according to the FSIS directive 10,250.1 [29], ensuring that all surfaces were thoroughly rinsed. The rinsate was then collected in a sterile labeled sample bottle for further testing.

### 2.3. Salmonella Isolation, Identification, and Quantification

To isolate *Salmonella* from the rinsate, a 60 mL aliquot was added into a Whirl-Pak and incubated at 42 °C for 24 h. After incubation, 1 mL of each incubated sample was added to 10 mL Rappaport Vassiliadis broth (RV) and Tetrathionate broth (TT) and then incubated at 42 °C for 24 h. The enriched samples were then streaked onto xylose lysine tergitol-4 (XLT4) agar and incubated at 37 °C for 24 h. After incubation, isolated presumptive *Salmonella* colonies were randomly selected and re-streaked on tryptic soy agar. Re-streaked colonies were confirmed in our laboratory using serum agglutination with BD Difco™ *Salmonella* Antiserum Poly A–I & VI and further serotyped. Isolated bacteria cultures were stored in glycerol at −80 °C for further analysis. Additionally, the samples were screened and quantified for *Salmonella* using BAX^®^ System SalQuant™ (Hygiena, Fresno, CA, USA), a commercial PCR-based system. Briefly, a 30 mL rinsate was added to 30 mL pre-warmed MP media placed in a Whirl-Pak and then incubated at 42 °C for intervals of 6 h and 24 h. After the appropriate incubation, *Salmonella* lysate (lysis buffer and protease) was prepared and added to each sample, and the samples were evaluated according to the BAX^®^ Real-Time *Salmonella* assay following the manufacturer’s protocol.

### 2.4. Campylobacter Isolation, Quantification, and Identification

For each sample, 30 mL of rinsate was enriched in 30 mL double-strength blood-free Bolton’s broth (2X-BFBB) supplemented with Oxoid™ Bolton’s broth selective supplement and incubated at 42 °C for intervals of 20 and 48 h under microaerophilic conditions. After 20 h incubation, samples were assessed with BAX^®^ Real-Time *Campylobacter* assay (Hygiena, Fresno, CA, USA) according to the manufacturer’s guidelines. Following 48 h incubation, samples were streaked onto *Campylobacter* agar and incubated at 42 °C for 48 h under microaerophilic conditions. Typical colonies exhibiting morphology for *Campylobacter* species were identified and stored as previously described. These colonies were further confirmed using BAX^®^ Real-Time *Campylobacter* assay.

### 2.5. Detection of Indicator and Spoilage Organisms

All samples were further evaluated for microbial indicators for contamination and spoilage, including aerobic bacteria count (AC), Enterobacteriaceae (EB), and lactic acid bacteria (LAB) using Neogen^®^ Petrifilm^®^ (Neogen, Lansing, MI, USA). One mL of each chicken rinsate was serially diluted in phosphate-buffered saline (PBS) and each dilution was then plated on AC, EB, and LAB Petrifilms™ and incubated at 37 °C for 48 h according to the manufacturer’s instructions. Colonies were enumerated, log-transformed, and reported in Log_10_ CFU/mL.

### 2.6. Antimicrobial Susceptibility Testing

A total of 22 *Salmonella* isolates (11 each from CON and RWA sources) and 24 *Campylobacter* isolates (CON = 11 and RWA = 13) were selected based on the serotypes and species identified in this study. These isolates were subjected to antimicrobial susceptibility testing following the NARMS (National Antimicrobial Resistance Monitoring System) guidelines [30]. The *Salmonella* isolates were tested against a panel of 13 antibiotics, while the *Campylobacter* isolates were tested against 8 antibiotics (Table 1). *Escherichia coli* ATCC 25,922 was included as an internal control strain to ensure the accuracy and reliability of results. The susceptibility testing was carried out using the Sensititre™ Complete Automated AST system (Thermo Fisher Scientific Inc., Waltham, MA, USA) following the manufacturer’s guidelines. Briefly, *Salmonella* isolates were suspended in demineralized water to achieve a 0.5 McFarland standard cell suspension. From this adjusted suspension, 10 µL inoculum was transferred into 11 mL Mueller–Hinton broth and vortexed thoroughly. The inoculum was dispensed into CMV5AGNF plates, with 50 µL added to each well of a 96-well plate containing antibiotics at different concentrations using the Sensititre AIM. The plates were sealed and incubated at 37 °C for 18–24 h. For *Campylobacter* isolates, suspensions prepared in 5 mL Mueller–Hinton broth were adjusted to a 0.5 McFarland standard. A volume of 100 μL of this suspension was then added to 11 mL Mueller–Hinton broth with laked horse blood and dispensed into CAMPY2 plates. The plates were sealed and incubated under microaerophilic conditions at 37 °C for 48 h. All plates were read using the Sensititre Vizion and the results were interpreted as susceptible, intermediate, or resistant according to NARMS breakpoints based on CLSI guidelines [30,31]

### 2.7. Statistical Analysis

All the pathogen incidence and indicators data were analyzed using SAS version 9.4 (SAS Institute Inc., Cary, NC, USA) [32]. The incidence of *Salmonella* and *Campylobacter* between the production systems, product types, and brands as well as the comparison of indicators (AC, EB and LAB) in Log_10_ CFU/mL were examined with ANOVA using the General Linear Model (GLM). Means were separated using Tukey’s HSD test, with significant level set at *p* ≤ 0.05. For quantification, BAX^®^ Hygiena SalQuant^®^ and CampyQuant™ methodologies were used to convert *Salmonella* and *Campylobacter* CT values to Log_10_ CFU/mL with a respective limit of quantification (LOQ) of 1 CFU/mL and 10 CFU/mL.

## 3. Results

The incidence and quantity of *Salmonella* and *Campylobacter* isolated from retail chicken (WOG, parts, and giblets) of CON and RWA production systems from brands (A and B) are shown in Table 2.

### 3.1. Incidence and Quantity of Salmonella in Retail Chicken

A total of 170 chicken samples were collected as follows: WOG (60); CON (A = 20) and RWA (A = 20 and B = 20), parts (80); 40 each for CON and RWA (20 each from brands A and B), and giblets (CON = 30). Across all samples, the overall incidence of *Salmonella* was 36% (62/170). The incidence of *Salmonella* was dynamic among all the parameters evaluated, i.e., production systems, product types, and brands. Between the production systems, *Salmonella* incidence in CON (33%, 20/60) was not different (*p* = 0.13) from RWA (23%, 18/80). However, a significant difference (*p* < 0.001) exists between the product types (WOG and parts) from CON and RWA systems with WOG (CON 60% (12/20) and RWA 38% (15/40)) having a higher incidence than parts (CON 20% (8/40) and RWA 8% (3/40)). When considering the brands for both product types (Table 2), there was no difference (*p* = 0.22), although for WOG, CON-A had the highest incidence with 60% followed by RWA-A at 50% and RWA-B at 25%; while parts had a generally lower incidence of 15% CON-A, 25% CON-B, 5% RWA-A, and 10% RWA-B. It is important to note that the absence of data for CON-B WOG in this study substantially reduced complete data comparison. The highest incidence of *Salmonella* was observed in giblets, where 80% of the samples collected were positive.

The quantity of *Salmonella* in the samples was remarkably low (Table 2). The positive samples from CON had values ranging from < 1 Log_10_ CFU/mL to 2.42 Log_10_ CFU/mL, while RWA had values that ranged from < 1 Log_10_ CFU/mL to 0.98 Log_10_ CFU/mL. Within the product types, WOG (CON-A) had the highest quantity at 2.42 Log_10_ CFU/mL, while RWA-A, being the highest for RWA was 0.86 Log_10_ CFU/mL. Importantly, RWA-B was below the LOQ. For parts, all the positive samples were below the LOQ except RWA-B at 0.98 Log_10_ CFU/mL. Giblets had the highest quantity ranging from < 1 Log_10_ CFU/mL to 3.43 Log_10_ CFU/mL. Notably, 38% (9/24) of the positive samples were below the LOQ while the remaining 63% (15/24) averaged at 1.01 Log_10_ CFU/mL.

#### Serogroup Distribution of *Salmonella* Isolates

**Figure 2:**
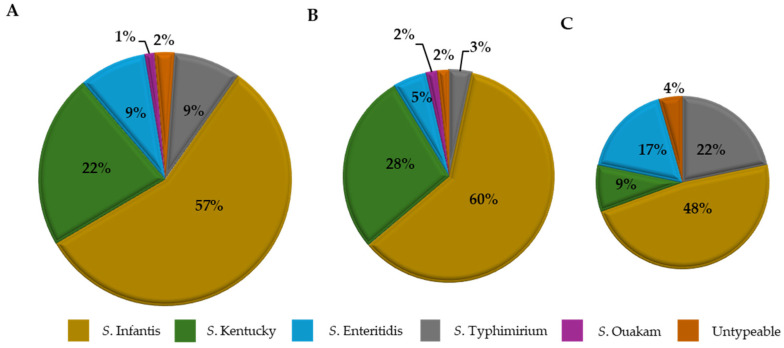
Distribution of *Salmonella* serotypes in the retail chicken (N = 170) samples from conventional (CON) and raised without antibiotics claim (RWA) sources. A total of 81 *Salmonella* isolates were recovered. (**A**) is the overall serotype distribution of 81 isolates, (**B**) is the distribution of the 58 (72%) isolates from CON source, and (**C**) is the distribution of the remaining 23 (28%) isolates from the RWA source.

Figure 2A–C shows the serotype distribution of *Salmonella* isolates based on the two production systems. Overall, 81 *Salmonella* isolates were recovered from all the chicken samples tested (n = 170) across both production systems. Of these isolates, a higher incidence of 72% (58/81) was from CON samples, while 28% (23/81) was RWA samples. A total of five *Salmonella enterica* serotypes were identified, and among the CON isolates, serotype Infantis at 60% (35/58) was the most prevalent followed by Kentucky at 28% (16/58), while serotypes Enteritidis 5% (3/58), Typhimurium 3% (2/58), and Ouakam 2% (1/58) were relatively low (Figure 2B). Similarly, for RWA isolates, serotype Infantis 48% (11/23) remained the most abundant, followed by serotypes Typhimurium 22% (5/23), Enteritidis 17% (4/23), and Kentucky 9% (2/23). Also, 2% (1/58) and 4% (1/23) from CON and RWA isolates were not typeable but serogrouped and belonged to the C_1_ group (Figure 2C).

### 3.2. Incidence and Quantity of Campylobacter in Retail Chicken

Overall, *Campylobacter* was found in 35% (59/170) of the chicken samples. The incidence of *Campylobacter* in CON samples at 25% (15/60) was not different (*p* = 0.58) from RWA 29% (23/80). However, differences exist (*p* < 0.0001) between the product types in which WOG for CON was 50% (10/20) and RWA was 45% (18/40), while parts were 13% (5/40) each. Similarly, differences were observed between brands where for WOG, RWA-A at 65% was significantly higher than RWA-B at 25% and all the parts (CON-A = 0% and B = 25%, RWA-A = 5% and B = 20%) (Table 2). Furthermore, *Campylobacter* was detected in 77% (23/30) of giblet samples.

For quantification, *Campylobacter* BAX positive in the chicken samples were 26% (44/170). Among these, 18% (11/60) were from CON samples (WOG and parts). Of the positive CON samples, 27% (3/11) were below the LOQ, while the remaining 73% (8/11) had an average of 2.15 Log_10_ CFU/mL with values ranging between 1.19 Log_10_ CFU/mL and 4.66 Log_10_ CFU/mL. For RWA samples from WOG and parts, 13% (10/80) were positive with 40% (4/10) below the LOQ and 60% (6/10) having an average of 1.70 Log_10_ CFU/mL, with quantity ranging between 1.01 Log_10_ CFU/mL and 2.2 Log_10_ CFU/mL. Notably, all the part samples were BAX^®^ negative except for one CON-B sample at 1.50 Log_10_ CFU/mL. Giblets had 77% (23/30) positives. Of these, 26% (6/23) were below the LOQ, while the remaining 74% (17/23) had values ranging from 1.01 Log_10_ CFU/mL to 5.3 Log_10_ CFU/mL

#### Species Distribution of *Campylobacter* Isolates

**Figure 3:**
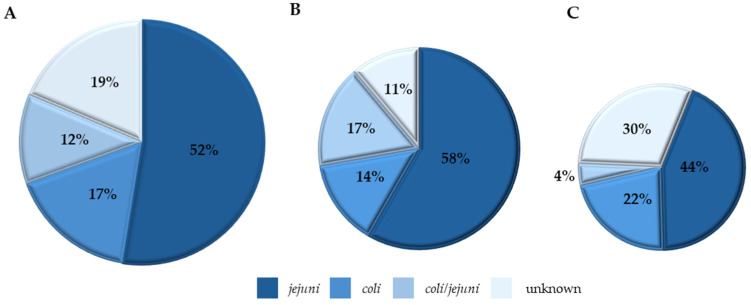
Distribution of *Campylobacter* species in the retail chicken (N = 170) samples from conventional (CON) and raised without antibiotics claim (RWA) sources. A total of 59 *Campylobacter* species recovered. (**A**) is the overall species distribution of 59 isolates, (**B**) is the distribution of the 36 (61%) isolates from the CON source, and (**C**) is the distribution of the remaining 23 (39%) isolates from the RWA source.

The species distribution of the *Campylobacter* isolates based on the production system is illustrated in Figure 3A–C. Overall, 59 *Campylobacter* were isolated from both production systems by culture methods and further identified using the BAX^®^ PCR system. Of the 59 culture-isolated pathogens, 61% (36/59) were detected in CON samples while 39% (23/59) were from RWA samples. In contrast, 44 *Campylobacter* isolates were identified using the BAX^®^ Real-Time *Campylobacter* assay which is based on a 20 h incubation period. From our results, the differences in the incidence of *Campylobacter* detected by culture method and BAX^®^ assay methods could be due to the differences in incubation periods. Specifically, 39% (23/59) of culture-positive isolates were BAX-negative, while 18% (8/44) of the BAX^®^ positive samples were culture-negative. *Campylobacter jejuni* and *coli* dominated the positive samples, but some samples were a mixture of both species at diverse relative abundance. *C. jejuni* was the most abundant in both CON and RWA samples. For CON samples, *C jejuni* was 58% (21/36), *C. coli* 14% (5/36), and mixed-species 17% (6/36); while 11% (4/36) were not identified because the isolates could not be revived after storage. RWA samples were also characterized as *C. jejuni* 43% (10/23), *C. coli* 22% (5/23), mixed-species 4% (1/23), and 30% (7/23) could not be revived/identified due to storage conditions.

### 3.3. Indicator and Spoilage Organisms

**Figure 4:**
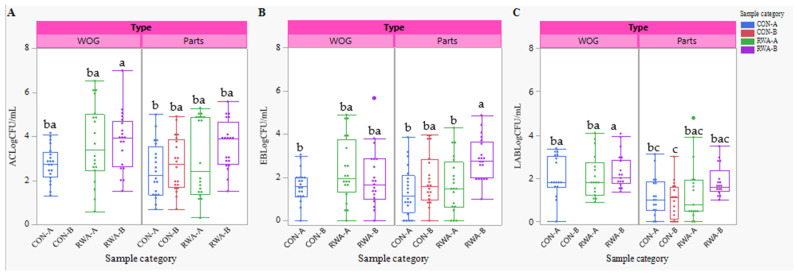
Incidence of Indicator organisms: (**A**) Aerobic Count–AC, (**B**) Enterobacteriaceae–EB, and (**C**) Lactic acid bacteria in Log_10_ CFU/mL of WOG and Parts from CON and RWA sources. Mean Log_10_ CFU/mL with different lowercase letters indicate significant differences. Superscripts are omitted where differences were not statistically significant (*p* > 0.05).

The average aerobic count (AC), Enterobacteriaceae (EB), and lactic acid bacteria (LAB) counts, expressed as Log_10_ CFU/mL in the chicken samples (WOG, parts, and giblets) from both production systems (CON and RWA) and brands (A and B) are illustrated in Figure 4A–C. These indicators are essential for assessing process control during post-harvest processing to prevent the incidence of foodborne illness.

The AC count between the CON and RWA for WOG and parts differed significantly (*p* = 0.0003) with CON having an average count of 2.63 Log_10_ CFU/mL and 3.52 Log_10_ CFU/mL for RWA. However, the AC counts between the product types (WOG and parts) and brands were not different (*p* = 0.39). In the WOG samples, the average (standard deviation) count for CON-A was 2.73 (0.79) Log_10_ CFU/mL, whereas, RWA-A and RWA-B had a higher count of 3.66 (1.77) and 3.79 (1.31) Log_10_ CFU/mL, respectively. Similarly for parts, the average counts for CON-A and CON-B were lower at 2.45 (1.24) Log_10_ CFU/mL and 2.72 (1.24) Log_10_ CFU/mL, respectively, while RWA-A and RWA-B were higher at 3.04 (1.79) and 3.61 (1.11) Log_10_ CFU/mL, respectively (Figure 4A). The AC count in giblets was the highest at an average of 4.97 (1.09) Log_10_ CFU/mL.

Similar to AC, the production systems differed significantly (*p* = 0.005) for EB count with an average count of 1.63 Log_10_ CFU/mL for CON and 2.21 Log_10_ CFU/mL for RWA. Between the brands, for WOG the average count and standard deviations for CON-A, RWA-A, and RWA-B were 1.61 (0.72), 2.33 (1.49), and 1.97 (1.35) Log10 CFU/mL, respectively, whereas for parts CON-A and CON-B they were 1.36 (1.15) and 1.91 (1.19) Log_10_ CFU/mL, respectively, and for RWA-A and RWA-B they were 1.68 (1.28) and 2.86 (0.98) Log_10_ CFU/mL, respectively. Importantly, a significant difference (*p* = 0.006) was observed between RWA-B and the other product types except for RWA-A (WOG) (Figure 4B).

The lactic acid bacteria count differed between the production systems (*p* = 0.03) and product types (*p* = 0.0002). CON samples had an average count of 1.44 Log_10_ CFU/mL while RWA samples had 1.92 Log_10_ CFU/mL. Among the product types and brands, WOG (CON-A, RWA-A, and RWA-B) had average counts of 2.10 (0.93), 2.09 (1.00), and 2.34 (0.82) Log_10_ CFU/mL, respectively. For parts samples, the respective counts were 1.21 (0.90) and 1.01 (0.84) Log_10_ CFU/mL (CON-A, CON-B) and 1.40 (1.38) and 1.85 (0.71) Log_10_ CFU/mL (RWA-A and RWA-B) (Figure 4C). Giblets remained the highest with an average count of 3.42 Log_10_ CFU/mL.

### 3.4. Antimicrobial Susceptibility Profile of Selected Salmonella and Campylobacter Isolates

**Figure 5:**
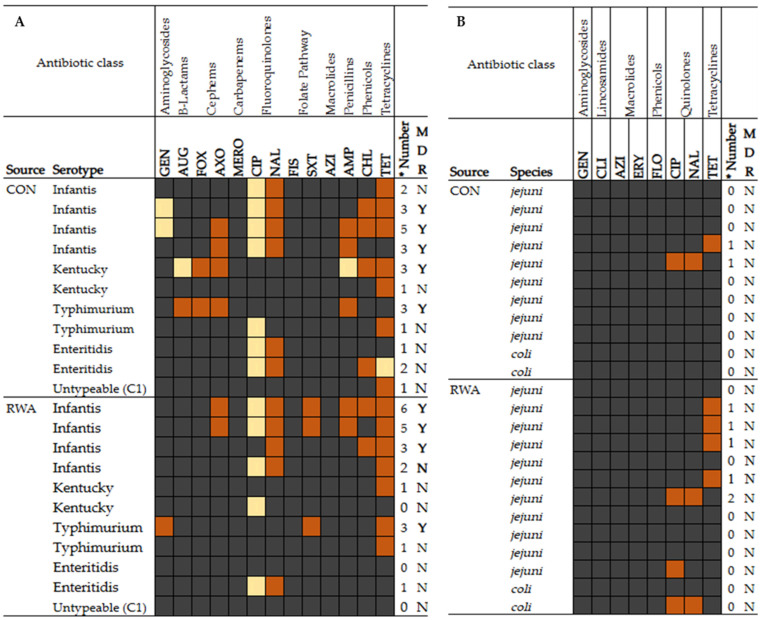
Heatmap showing the antibiotic susceptibility pattern of *Salmonella* (**A**) and *Campylobacter* (**B**) isolated from retail chicken samples. Antibiotic susceptibility is classified according to the CLSI and NARMS guidelines. Dark grey bar represents susceptible (S), yellow bar is intermediate (I), and red bar is resistant (R) for each antibiotic. Abbreviations and concentrations of antimicrobial agents are listed in Table 1. Multi-drug resistance (MDR) is defined by NARMS guidelines as possessing resistance to ≥3 classes of antimicrobials; * Number is the number of resistance classes. MDR classification is denoted with Y as yes or N as no. The serotypes and production source are specified for each isolate. CON is conventional and RWA is raised without antibiotics.

The antimicrobial susceptibility profile of the tested *Salmonella* and *Campylobacter* isolates was presented in Figure 5A,B. Out of the 22 *Salmonella* isolates tested (Figure 5A), 91% (20/22) were resistant to at least one antibiotic and 41% (9/22) exhibited multidrug (MDR) resistance. Of the MDR isolates, 45% (5/11) were from CON and 36% (4/11) were from RWA, showing that CON isolates displayed a greater antibiotic-resistant pattern compared to RWA isolates. The highest resistance in the isolates was to tetracycline-TET 64% (14/22) followed by nalidixic acid-NAL 50% (11/22), and the least was observed to amoxicillin–clavulanic acid-(AUG) at 5% (1/22) which is a CON isolate. All the isolates were susceptible to meropenem-MERO, sulfisoxazole-FIS, and azithromycin-AZI. Furthermore, all the CON isolates were susceptible to trimethoprim/sulfamethoxazole (SXT) while all RWA isolates were susceptible to amoxicillin–clavulanic acid–AUG and cefoxitin–FOX. Meanwhile, two isolates (RWA) were pan-susceptible to all the antibiotic tested.

Twenty-four *Campylobacter* isolates from both CON and RWA were tested against eight antibiotics and their susceptibility profiles are illustrated in Figure 5B. Overall, 21% (5/24) of CON and RWA isolates were resistant to tetracycline-TET, 17% (4/24) to ciprofloxacin-CIP, and 13% (3/24) nalidixic acid-NAL. Notably, all the isolates tested were susceptible to antibiotics in the aminoglycosides, lincosamides, macrolides, and phenicols classes. A total of 15 (63%) were pan-susceptible with 9/12 (82%) from CON and 6/12 (46%) from RWA.

## 4. Discussion

The poultry supply chain is highly complex, with the risk of bacterial contamination emerging at all stages of the production continuum, from pre-harvest (on farm) to post-harvest (processing), and retail (consumers) levels. However, the goal of a poultry integrator is to ensure food safety at all these stages. While the prevalence of foodborne pathogens in poultry remains a concern, the increased popularity of non-conventional systems against conventional systems is evident [25]. However, the correlation between these production systems and pathogen prevalence at pre-harvest, post-harvest, and retail remains inconclusive. Several studies have evaluated this scenario and reported variable findings. Novoa Rama et al. [33] revealed a higher *Salmonella* prevalence in conventional farms (73%) than in non-conventional (7%) during early production, Poudel et al. [34] indicated that the presence of *Campylobacter* at a non-conventional farm (31.4%) is like a conventional farm (40%). Furthermore, a lower incidence of *Campylobacter* in non-conventional farms (45%) as against conventional (73%) was reported by [35]. At post-harvest, numerous studies [36,37,38] have also compared the prevalence of these pathogens in conventional and non-conventional processing plants, with findings revealing varying results. Moreover, a comprehensive study on pathogen contamination throughout both conventional and alternative poultry supply chains collecting over a hundred thousand datasets across the production chain further confirmed this variability [25]. Unlike pre-harvest and post-harvest, some of the most cited studies [23,24,26] on the incidence of these pathogens in retail chicken products in the United States are limited and only goes back to 2005 and 2014, but recent studies from [22,27] have provided significant updates on the impact of production systems on *Salmonella* at the retail level and the antibiotic resistance of pathogens from retail products. Given the increased incidence of foodborne outbreaks, recalls, and illnesses due to various foodborne pathogens reported in 2024, there is a dire need for continuous surveillance information on retail food safety particularly for chicken products regardless of the production system. With this knowledge, stakeholders can focus on reducing the levels of these pathogens from farm to fork.

### 4.1. Pathogen Incidence, Quantification, and Serogroup/Species Dynamics

In this present study, we evaluated the incidence of *Salmonella* and *Campylobacter* in retail chicken samples from CON and RWA production systems, and to ensure a broader scope, we examined different brands of chicken samples from the two production systems. The result of this study established the presence of *Salmonella* (36%) and *Campylobacter* (35%) in the chicken samples which align with reports from NARMS [39], emphasizing that chicken remains an important source of these foodborne pathogens. While both pathogens were present in the chicken samples, *Salmonella* incidence was higher in CON (33%) than RWA (23%). On the other hand, *Campylobacter* was lower in CON (25%) than RWA (29%). However, these differences were not statistically significant. Our findings align with reports from other studies [24,27,34]. For instance, Ref. [24] reported a *Salmonella* incidence of 22% in conventional and 21% in non-conventional chicken, with no significant difference, similar to our results. Meanwhile, Ref. [27] reported 62% and 37% in non-conventional and conventional, respectively, with statistically significant differences. Contrarily, some other studies like [23] reported higher *Salmonella* prevalence in non-conventional chicken while [34] found *Campylobacter* to be higher in conventional (40%) than non-conventional (31.4%) chicken. The incidence by product type showed that *Salmonella* and *Campylobacter* were higher in WOG for both CON and RWA than in parts. The low level of these pathogens in chicken parts can be attributed to further processing of the chicken parts and further antimicrobial intervention for cut-up meats. Notably, our study also evaluated incidence within brands of CON and RWA and found no major difference in *Salmonella* incidence. However, differences were observed for *Campylobacter*, which could be due to processing variations. Giblets consist of gizzard, liver, heart, and sometimes neck [40], which are all prone to bacteria contamination during processing because these parts are harvested during the evisceration process. Of all the chicken samples we evaluated, the highest incidence of both pathogens was found in the giblets. In addition to determining the incidence of these pathogens, unlike most retail chicken studies that focus solely on the incidence data, our study goes further by quantifying the loads of *Salmonella* and *Campylobacter* in the positive samples. Our results show a remarkably low level of *Salmonella* in the chicken samples. Quantifying the levels of these pathogens is critical for several reasons. The mere presence of pathogens does not always correlate with the risk of infection; the pathogen load according to the infectious dose and the susceptibility of individual consumer plays a significant role in determining the likelihood and severity of illness, and higher levels of pathogens in food products like chicken can increase the risk of contamination if cooking temperature is not accurately measured; therefore, proper handling and thorough cooking remain essential to reducing foodborne illness risks [41].

Understanding the serotypes and species of pathogens like *Salmonella* and *Campylobacter* across the poultry chain has helped identify and trace outbreaks [42]. Most isolates from CON and RWA recovered in this study belong to *Salmonella* serotypes commonly found in poultry, namely Infantis, Kentucky, Enteritidis, Typhimurium, and Ouakam. The serotypes identified in this study correspond to those frequently linked to foodborne outbreaks reported by NARMS in their 2015 report [43]. Moreover, USDA listed *Salmonella* Infantis as an increased trend in chicken [44]. The prevalence of these serotypes is not just found in retail chicken but has been traced back to farms and processing plants. Studies have reported the prevalence of these serotypes in poultry farms and processing environments [45,46,47,48]. Furthermore, in both production systems *Salmonella* Infantis was the most abundant and this finding is consistent with other studies that showed a higher prevalence of serotypes like Infantis [22,27]. *Campylobacter* has also been implicated in several outbreaks with *C. jejuni* and *C. coli* being the most common species found in poultry, accounting for nearly 86% of the reported cases in the United States [49]. These species were the two identified in our samples.

### 4.2. Indicators and Lactic Acid Bacteria

Indicator organisms, such as aerobic bacteria, Enterobacteriaceae (EB), and lactic acid bacteria (LAB), are used to assess hygiene quality, the likelihood of bacterial contamination, and food quality. Aerobic counts represent the overall measure of microbial load and environmental contamination, while EB is more specific to fecal contamination or inadequate hygiene control [50]. A high EB count often indicates the presence of *Salmonella* or other Enterobacteriaceae in a sample and could be used to assess the effectiveness of control measures without testing for *Salmonella.* The presence of lactic acid bacteria in food is not a food safety concern but is often used to assess spoilage and deterioration. Until this study, there was limited information on the comparative analysis of indicators and spoilage organisms of retail chicken in conventional and non-conventional poultry sources. Comparing CON and RWA, the AC in CON was slightly lower than that of RWA. Similar trends were observed for chicken parts, but giblets consistently exhibited the highest AC counts. Interestingly, lactic acid bacteria count showed little variation across production systems and brands. LAB counts in whole chicken samples averaged 2.22 Log_10_ CFU/mL while chicken parts exhibited slightly lower counts. It is important to note that the low LAB count across all the chicken samples can be attributed to good storage conditions at the stores which could help limit bacteria growth, offering consumers chicken products that are free of food safety and quality concerns.

### 4.3. Antimicrobial Susceptibility

The antimicrobial resistance (AMR) of pathogens associated with poultry remains a public health concern. According to the CDC, the recommended antibiotics for *Salmonella* infections in humans include fluoroquinolones like ciprofloxacin for adults, azithromycin for children, and third-generation cephalosporins like ceftriaxone as an alternative first-line treatment option [51,52]. In this study, 91% of the selected 22 *Salmonella* isolated from all the chicken samples were resistant to at least one antibiotic and 41% exhibited resistance to three or more drugs. As expected, the prevalence of multidrug resistance (MDR) *Salmonella* was lower in RWA poultry samples compared to CON samples. This is likely due to differences in historic antibiotic usage under the two production systems. Studies have mentioned that the prophylactic and therapeutic use of antibiotics are the main drivers of AMR and MDR in poultry production systems [51]. The current study report is similar to recent findings from NARMS, which showed 32% of retail chickens being multidrug-resistant [39]. The highest resistance in the current study was to tetracycline (64%) followed by nalidixic acid (50%) aligning with other studies across poultry production [27,53] that have reported tetracycline as the most common resistant antibiotic in *Salmonella* isolates. These studies found similar patterns to our study, with [27] reporting 82.8% of the isolates evaluated showed resistance to tetracycline and similarly [53] reported 72% resistance to tetracycline. Contrarily, Ref. [54] reported that non-conventional isolates exhibited lower resistance to tetracycline. The observed high resistance of nalidixic acid raises significant public health concerns because the antibiotics are from the drug class fluoroquinolones, listed by the CDC as the drug of choice to treat *Salmonella* infections. *Salmonella* serotypes in this study showed varying resistance pattern and serotype Infantis exhibited the most MDR, which is consistent with [27] that reported similar findings. This is concerning because [27] reported *S*. Infantis as emerging but findings as far back as 2017 indicated that it was mostly reported as pan-susceptible with only an occasional MDR phenotype [55]. These results indicate the need for additional investigations into the evolving resistance pattern. Notably, the low resistance rates to amoxicillin–clavulanic acid and susceptibility to meropenem, sulfisoxazole, and azithromycin are encouraging because these antibiotics remain viable treatment options.

Like *Salmonella*, *Campylobacter* resistance trends are critical. NARMS data show that *C. jejuni* and *C. coli* isolates commonly exhibit resistance to fluoroquinolones and macrolides, which are critical antibiotics for treating campylobacteriosis [34,49]. From this study, isolates were only resistant to three antibiotics–ciprofloxacin, nalidixic acid, and tetracycline–and this is consistent with findings by [49] who found resistance to these antibiotics in pre-harvest (farm) isolates. Resistance to these antibiotics aligns with global trends and raises concerns given the clinical importance of these antibiotics. It is noteworthy that all the isolates were susceptible to the drug class of aminoglycosides, lincosamides, macrolides, and phenicol; however, the resistance pattern (TET > CIP > NAL) displayed by some of the isolates remains a concern for campylobacteriosis treatment [56]. One of the limitations to the AMR examination of the isolated pathogens in this study is the use of a small set of *Salmonella* and *Campylobacter* from CON and RWA sources, limiting our ability to make inferences about the resistance patterns and correlate that to the production systems. To address this limitation, a more comprehensive investigation into antimicrobial resistance in *Salmonella* from these two sources along the poultry production continuum is ongoing.

## 5. Conclusions

The presence of *Salmonella* and *Campylobacter* in retail chicken samples from the two production systems emphasizes the challenges posed by these foodborne pathogens for poultry producers. Contrary to consumers’ perceptions of conventional and non-conventional poultry meat, our findings reveal no major differences in pathogen prevalence between conventional and non-conventional systems, highlighting that production methods have minimal impact on pathogen levels. Although these pathogens were detected in the retail chicken samples, an average of 49% of the positive samples were below the detection limit; therefore, with appropriate storage conditions, cooking, and handling practices, the risk of contamination can be effectively minimized, ensuring food safety for consumers. However, the high microbial loads observed in the aerobic count for all the samples, particularly giblets, highlight the need for stricter process monitoring and control to further reduce contamination risks both from pathogenic and opportunistic bacteria. Continued effort from farm to fork is essential, with all stakeholders playing a critical role in reducing *Salmonella* and *Campylobacter* contamination in chicken and foodborne illness in consumers. Producers should put more effort into further minimizing pathogen contamination and controlling the emergence of antibiotic-resistant strains. Also, consumers must be mindful of the risks posed by deviating from food safety practices like proper storage, proper handling, and appropriate cooking. This study provides valuable insights into pathogen incidence, loads, serotype/species dynamics, and antimicrobial resistance in retail chicken, emphasizing the need for continued monitoring, intervention, and evaluation of food safety practices throughout the poultry supply chain.

## Figures and Tables

**Table 1:** Antibiotics tested and range of concentrations according to NARMS guidelines.

CLSI Class	Antimicrobial Agent	Abbreviation	Range of Concentration (μg/mL)
*Salmonella* isolates			
Aminoglycosides	Gentamicin	GEN	0.25–16
β–lactam agents	Amoxicillin-clavulanic acid	AUG	1/0.5–32/16
Cephems	Cefoxitin	FOX	0.5–32
	Cephems	AXO	0.25–64
Folate pathway antagonists	Sulfisoxazole	FIS	16–256
	Trimethoprim/Sulfamethoxazole	SXT	0.12/2.38–4/76
Macrolides	Azithromycin	AZI	0.25–32/0.12–16
Penems	Meropenem	MERO	0.06–4
Penicillins	Ampicillin	AMP	1–32
Quinolones	Ciprofloxacin	CIP	0.015–4
	Nalidixic acid	NAL	0.5–32
Phenicols	Chloramphenicol	CHL	2–32
Tetracyclines	Tetracycline	TET	4–32
*Campylobacter* isolates			
Aminoglycosides	Gentamicin	GEN	0.25–16
Lincosamides	Clindamycin	CLI	0.03–16/0.016–256 *
Macrolides	Azithromycin	AZI	0.015–64/0.016–256 *
	Erythromycin	ERY	0.03–64/0.016–256 *
Phenicols	Florfenicol	FLO	0.03–64
Quinolones	Ciprofloxacin	CIP	0.015–64/0.002–32 *
	Nalidixic acid	NAL	4–64/ 0.016–256 *
Tetracyclines	Tetracycline	TET	0.06–64/0.016–256 *

* E-test dilution range used before 2005.

**Table 2:** *Salmonella* and *Campylobacter* incidence (%) and quantity (Log_10_ CFU/mL) in retail chicken samples from different brands of conventional (CON) and raised without antibiotics (RWA) sources.

Product Type	Production System	* Brand	n	*Salmonella* Positives	*Salmonella* Quantity Range Log_10_ CFU/mL	*Campylobacter* Positives	*Campylobacter* Quantity Range Log_10_ CFU/mL
WOG	CON	A	20	12 (60) ^a^	<1–2.42	10 (50) ^ba^	10 (<10–4.66)
		B	-	-	-	-	
	RWA	A	20	10 (50) ^ba^	<1–0.86	13 (65) ^a^	10 (<10–2.2)
		B	20	5 (25) ^bac^	<1	5 (25) ^bc^	-
Parts	CON	A	20	3 (15) ^bc^	<1	0 ^c^	-
		B	20	5 (25) ^bac^	<1–0.28	5 (25) ^bc^	1 (1.5)
	RWA	A	20	1 (5) ^c^	<1	1 (5) ^c^	-
		B	20	2 (10) ^c^	0.98	4 (20) ^bc^	-
Giblets	CON	A	30	24 (80)	<1–3.43	21 (70)	23 (<10–5.3)

* Letters A and B denote different brands for each product type. Percentage values followed by different lowercase letters indicate significant differences. Superscripts are omitted for comparisons where differences were not statistically significant (*p* ≥ 0.05).

## Data Availability

The original contributions presented in the study are included in the article, further inquiries can be directed to the corresponding author.
